# Æsculapian Society of the Wabash Valley

**Published:** 1878-02

**Authors:** Chas. B. Johnson

**Affiliations:** Sec’y.; Tolono, Ills.


*Meeting of Æsculapian Society of Wabash Valley.*

The thirty-first annnal session of the Æsculapian Society of the Wabash Valley was held at Paris, Ills., on the 21st and 22d days of November, 1877.

The Society was called to order at 10.30 a. m. by the President, Dr. G. T. Ragan, of Neoga. Prayer was offered by the Rev. E. D. Wilkin, after which the society listened to a brief address of welcome from Dr. Wm. Massie in behalf of the local physicians.

At 2 p. m. was presented the report of the Chairman of the Committee on Obstetrics (Dr. O. C. Tobey, of Westfield) on post partum hemorrhage and rupture of the perineum.

The histories and treatment of several interesting cases were detailed. The paper was discussed by Drs. Rowe, Mosely and Mitchell.

Dr. J. L. Polk, of Arcola, next read a paper on post partum hemorrhage. The author held that ergot administered immediately after the birth of the child and continued for a time was a reliable prophylactic of hemorrhage. The paper was discussed by Drs. Massie, McKown, Barton, Cannon and others. Dr. Barton approved of the treatment advocated in the paper, but would give much larger doses of ergot than the writer recommended; would not hesitate to give four drachms, at a dose, of the fluid extract should the emergency require energetic treatment.

At 7.30 p. m. Dr. G. T. Ragan delivered his valedictory address at the M. E. church.

At the regular evening session Dr. Geo. Calloway read a paper on pneumonia and Dr. L. L. Silverthorn reported a case in which all the symptoms of pneumonia yielded in 48 hours after the exhibition of 20 grains of salicylic acid every two hours.

The election of officers for the ensuing year resulted as follows: President, Dr. J. M. McKown, of Arcola; Vice President, Dr. L. L. Silverthorn, of Charleston; Secretary and Treasurer, Dr. C. B. Johnson, of Tolono; Board of Censors, Drs. Baum, Barton, A, T. Steele, W. M. Chambers, jr., and Polk.

On Thursday morning, November 22, Dr. J. P. Worrel made a report on the treatment of the more common diseases of the ear.

Dr. James, of Greenup, presented an interesting pathological specimen—a fœtus obtained post mortem after extra-uterine pregnancy of three years standing.

After the appointment of the usual committees and the transaction of some miscellaneous business, the society adjourned to meet at Mattoon, Ills., on the last Wednesday in May, 1878.

CHAS. B. JOHNSON, M. D., *Sec'y.*

TOLONO, ILLS.

